# Exploring potential mediators of the cardiovascular benefit of dulaglutide in type 2 diabetes patients in REWIND

**DOI:** 10.1186/s12933-021-01386-4

**Published:** 2021-09-25

**Authors:** Manige Konig, Matthew C. Riddle, Helen M. Colhoun, Kelley R. Branch, Charles M. Atisso, Mark C. Lakshmanan, Reema Mody, Sohini Raha, Hertzel C. Gerstein

**Affiliations:** 1 Eli Lilly and Company, Lilly Corporate Center, Indianapolis, IN 46285 USA; 2 Oregon Health & Science University, Portland, USA; 3 University of Edinburgh, Edinburgh, UK; 4 University of Washington, Seattle, USA; 5 McMaster University, Hamilton, ON Canada

**Keywords:** Cardiovascular, Diabetes, Dulaglutide, Glucagon-like peptide-1, Mediators

## Abstract

**Background:**

The REWIND trial demonstrated cardiovascular (CV) benefits to patients with type 2 diabetes and multiple CV risk factors or established CV disease. This exploratory analysis evaluated the degree to which the effect of dulaglutide on CV risk factors could statistically account for its effects on major adverse cardiovascular events (MACE) in the REWIND trial.

**Methods:**

Potential mediators of established CV risk factors that were significantly reduced by dulaglutide were assessed in a post hoc analysis using repeated measures mixed models and included glycated hemoglobin (HbA1c), body weight, waist-to-hip ratio, systolic blood pressure, low-density lipoprotein (LDL), and urine albumin/creatinine ratio (UACR). These factors, for which the change in level during follow-up was significantly associated with incident MACE, were identified using Cox regression modeling. Each identified variable was then included as a covariate in the Cox model assessing the effect of dulaglutide on MACE to estimate the degree to which the hazard ratio of dulaglutide vs placebo was attenuated. The combined effect of the variables associated with attenuation was assessed by including all variables in an additional Cox model.

**Results:**

Although all evaluated variables were significantly improved by treatment, only changes in HbA1c and UACR were associated with MACE and a reduction in the effect of dulaglutide on this outcome was observed. The observed hazard ratio for MACE for dulaglutide vs placebo reduced by 36.1% by the updated mean HbA1c, and by 28.5% by the updated mean UACR. A similar pattern was observed for change from baseline in HbA1c and UACR and a reduction of 16.7% and 25.4%, respectively in the hazard ratio for MACE with dulaglutide vs placebo was observed. When HbA1c and UACR were both included, the observed hazard ratio reduced by 65.4% for the updated mean and 41.7% for the change from baseline with no HbA1c-UACR interaction (P interaction = 0.75 and 0.15, respectively).

**Conclusions:**

Treatment-induced improvement in HbA1c and UACR, but not changes in weight, systolic blood pressure, or LDL cholesterol, appear to partly mediate the beneficial effects of dulaglutide on MACE outcomes. These observations suggest that the proven effects of dulaglutide on cardiovascular disease benefit are partially related to changes in glycemic control and albuminuria, with residual unexplained benefit.

Clinicaltrials.gov; Trial registration number: NCT01394952. URL: https://clinicaltrials.gov/ct2/show/NCT01394952

## Background

Outcome trials evaluating cardiovascular (CV) effects of glucagon-like peptide-1 (GLP-1) receptor agonists have consistently demonstrated the benefit and safety of these agents in populations with type 2 diabetes (T2D) [1–7]. Some of these agents have significantly reduced major adverse cardiovascular event (MACE) outcomes, prompting revisions to the placement of such treatments in the guidance algorithms from professional societies [8–11]. The mechanisms through which GLP-1 receptor agonists exert these beneficial effects are not completely understood. Recognized effects of these agents that might contribute to improved CV outcomes include improvements in glycemic control, reduction in weight and adiposity, reduction in blood pressure, improved lipid profiles, and reduction in diabetes-associated complications including renal disease [12–14].

The Researching cardiovascular Events with Weekly INcretin in Diabetes (REWIND; NCT01394952) trial showed that, in patients with T2D and multiple CV risk factors or established CV disease, dulaglutide 1.5 mg once weekly reduced the incidence of a composite outcome comprising nonfatal myocardial infarction, nonfatal stroke, or death from CV causes or unknown causes (MACE) compared to placebo, with an observed hazard ratio (HR) of 0.88 (95% CI 0.79, 0.99; p = 0.026) [15]. The trial also demonstrated beneficial effects of dulaglutide on glycated hemoglobin (HbA1c), body weight, waist-to-hip ratio (WHR), systolic blood pressure (SBP), and low density lipoprotein (LDL) cholesterol [15]. Here, we report the results of an exploratory analysis evaluating these factors as potential mediators of the reduction in MACE.

## Methods

### Study design and participants

The REWIND study design and eligibility criteria have been published previously [15–17]. Briefly, people who were at least 50 years of age or older with type 2 diabetes, a HbA1c ≤ 9.5% on stable doses of up to 2 oral glucose lowering drugs with or without basal insulin, a prior CV event or at least 2 CV risk factors, and a body mass index ≥ 23 kg/m^2^ were included. Participants were randomly assigned to the addition of once weekly subcutaneous dulaglutide 1.5 mg or placebo and the usual standard of care (as informed by current country guidelines). Key exclusion criteria were an estimated glomerular filtration rate less than 15 mL/min per 1.73 m^2^, cancer in the previous 5 years, severe hypoglycemia in the previous year, life expectancy less than 1 year, a coronary or cerebrovascular event within the previous 2 months, and plans for revascularization. A complete list of inclusion and exclusion criteria is given in the appendix (pp 151–55) of the primary results paper [15].

### Statistical analysis

A mediation analysis approach [18] was used to estimate the degree to which the effect of one or more of the CV risk factors previously found to be favorably affected by dulaglutide could statistically account for its effect on the primary MACE outcome. These risk factors included HbA1c, LDL, body weight, SBP, WHR, and UACR. The statistical analysis involves the effect of these variables calculated in two ways, i.e., change from baseline and updated mean. Change from baseline is defined as the last observed change from baseline before the occurrence of MACE and the updated mean is defined as the mean value considering all the prior values of the variable before the occurrence of MACE. The effect of dulaglutide was re-estimated after including the updated mean and change from baseline for these CV risk factors during the entire period of observation in the trial.

The epidemiologic relationship between each of these variables and the primary MACE outcome was estimated by separate Cox models fitted using either (1) the updated mean value of the variable until a MACE occurred or the end of the period of observation, or (2) the change from baseline for the variable to the last measurement before an event or the end of observation. The only independent variable for each model was the variable being assessed. WHR analyses were performed separately for each sex because of known sex-related differences [15].

For those variables for which either the updated mean or the change from baseline was significantly associated with the primary MACE endpoint at a nominal p-value of 0.05, the hazard ratio for dulaglutide vs placebo of the primary MACE outcome was then re-estimated using a separate Cox model. The model included treatment group, the baseline value of the measurement, and the updated mean or the change from baseline of the variable, respectively as time-dependent covariates. These produced univariate models of the treatment effect adjusted for each potential mediator. The proportion of the effect of dulaglutide mediated by the variable was estimated by *100 x (ln HR*_*unadjusted*_* – ln HR*_*adjusted*_*))/ln HR*_*unadjusted*_ where ln(HR_unadjusted_) is the natural logarithm of the hazard ratio comparing dulaglutide to placebo from the Cox model incorporating only the treatment effect (i.e., the value from the primary analysis) [15], and ln(HR_adjusted_) is the natural logarithm of the hazard ratio from the model incorporating the potential mediator [19–25]. This estimate of mediation proportion effect and the 95% confidence intervals were computed and reported for both the change from baseline and updated mean of the variable separately.

All variables that were identified as possible mediators when analyzed individually were included together in a multivariable Cox model. Specifically, the hazard ratio of dulaglutide vs placebo on the primary MACE outcome was re-estimated after adjusting for the baseline value of each included variable, the change over time for each variable, and an interaction term for the included variables. As above, this was performed twice, using either the updated mean or the change from baseline of the variables. The proportion of treatment effect accounted for by these multivariable models was evaluated using the method described above for univariate models. SAS version 7.15 was used for all of the statistical analyses.

## Results

### Effects of dulaglutide on potential mediators over time

**Fig. 1:**
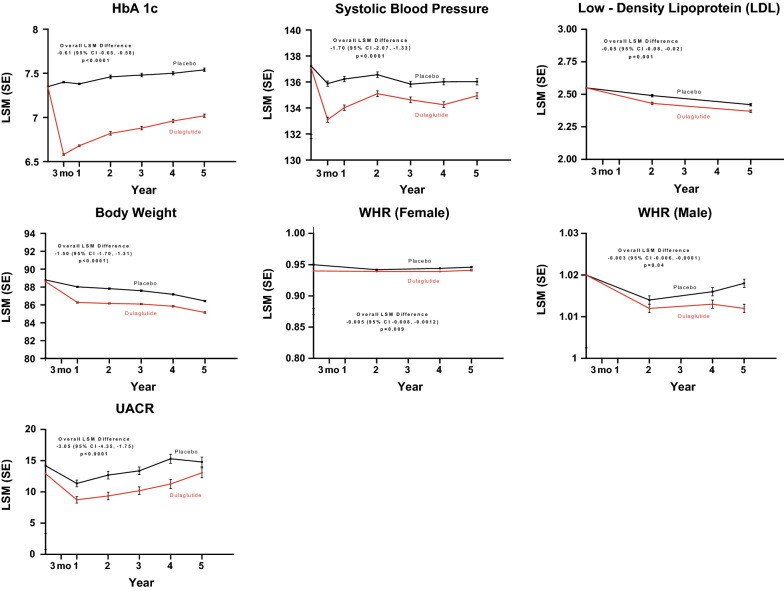
Effects of treatment on potential mediators over time up to 6 years: dulaglutide versus placebo. Dulaglutide significantly reduced HbA1c, body weight, WHR, SBP, LDL, and UACR over time from baseline compared to placebo. *HbA1c* hemoglobin A1c, *LDL* low density lipoprotein, *LSM* least squares mean, *SBP* systolic blood pressure, *SE* standard error, *UACR* urine albumin creatinine ratio, *WHR* waist-to-hip ratio

As previously reported, the REWIND trial recruited 9901 people (46.3% women) with type 2 diabetes whose mean age was 66.2 (SD 6.5) years. After random assignment to dulaglutide (N = 4949) or placebo (N = 4952), participants were followed for a median of 5.4 years during which 1257 individuals developed at least one MACE outcome [17]. The patterns of response of the CV risk factors that were favorably affected by assignment to dulaglutide versus placebo, together with the overall least squares mean between-treatment difference, are shown in Fig. 1.

The epidemiologic relationship between the change in each of these variables and incident MACE outcomes is shown in Table 1. Only two variables, the change in HbA1c and the change in UACR, were significantly associated with the MACE outcome using both the updated mean and the change from baseline to the last measurement. Thus, for HbA1c, the observed hazard ratio of the MACE outcome increased by 5% (HR 1.047, 95% CI 1.000, 1.008) for every 1% change in elevated HbA1c from baseline and by 9% (HR 1.087, 95% CI 1.033, 1.144) for every 1% higher elevated mean HbA1c. For UACR, the observed hazard ratio of the MACE outcome increased by 0.2% (HR 1.002, 95% CI 1.002, 1.003) for every mg/mmol increased change in UACR from baseline and by 0.3% (HR 1.003, 95% CI 1.002, 1.004) for every mg/mmol increased updated mean UACR.

**Table 1:** Biochemical measurements that potentially mediated the effect of dulaglutide on MACE-single factor analysis

	Potential mediators						
	HbA1c (%)^a^	Body weight (kg)	Systolic BP (mmHg)	LDL (mmol/L)	UACR (mg/mmol)^a^	WHR in men (m)	WHR in women (m)
Baseline value for dulaglutide^b^	7.3% (1.1)	88.5 (18.4)	137.1 (16.6)	2.56 (0.98)	1.80 (0.70–6.60)	1.02 (0.07)	0.94 (0.07)
Baseline value for placebo^b^	7.4% (1.1)	88.9 (18.6)	137.3 (17.0)	2.56 (0.98)	1.88 (0.70–7.38)	1.02 (0.07)	0.95 (0.07)
LSM change from baseline (CI)	− 0.61 (− 0.65, − 0.58)*	− 1.50 (− 1.70, − 1.31)*	− 1.70 (− 2.07, − 1.33)*	− 0.05 (− 0.08, − 0.02)*	− 3.05 (− 4.35, − 1.75)*	− 0.003 (− 0.006, − 0.000)*	− 0.005 (− 0.008, − 0.001)*
HR of updated mean value of variable on MACE	1.087 (1.033, 1.144)*	0.994 (0.983, 1.005)	0.999 (0.995, 1.004)	1.054 (0.958, 1.159)	1.003 (1.002, 1.004)*	1.876 (0.277, 12.721)	2.645 (0.295, 23.742)
HR of dulaglutide on MACE (after adjusting for the baseline and updated mean value of the variable)	0.923^c^ (0.819, 1.039)	0.889^c^ (0.784, 1.007)	0.880 (0.787, 0.984)	0.937^c^ (0.811, 1.082)	0.914^c^ (0.804, 1.039)	0.926^c^ (0.776, 1.104)	0.915^c^ (0.721, 1.160)
Percentage mediated by updated mean towards the effect of dulaglutide on MACE^d^	36.1	6.2	− 1.7	48.0	28.5	23.0	45.0
HR of change from baseline value of variable on MACE	1.047 (1.000, 1.008)*	0.998 (0.988, 1.008)	1.000 (0.996, 1.003)	1.064 (0.984, 1.151)	1.002 (1.002, 1.003)*	1.434 (0.310, 6.631)	1.828 (0.296, 11.284)
HR of dulaglutide on MACE (after adjusting for the baseline and change from baseline value of the variable)	0.900^c^ (0.801, 1.013)	0.896^c^ (0.791, 1.014)	0.880 (0.787, 0.984)	0.937^c^ (0.811, 1.083)	0.910^c^ (0.801, 1.035)	0.925^c^ (0.776, 1.103)	0.913^c^ (0.720, 1.158)
Percentage mediated by change from baseline toward the effect of dulaglutide on MACE^d^	16.7	12.3	− 1.5	48.6	25.4	22.3	44.2

*BP* blood pressure, *CI* confidence interval, *HR* hazard ratio, *IQR* interquartile rate, *LDL* low density lipoprotein, *LSM* least squares mean, *MACE* major adverse cardiovascular event, *SD* standard deviation, *UACR* urine albumin/creatinine ratio, *WHR* waist-to-hip ratio

^a^Variables that satisfy all 3 conditions to be a mediator

^b^The value is presented in mean (SD) or in median (IQR)

^c^The estimated HR less than the HR for the unadjusted model

^d^Compared to the previously reported HR of dulaglutide vs placebo on MACE of 0.882 (95% CI 0.789, 0.985) except for WHR (men) was compared with HR of dulaglutide on MACE for male subgroup of 0.905 (95%CI 0.787,1.040) and WHR (women) was compared with HR of dulaglutide on MACE for female subgroup of 0.850 (95% CI 0.709,1.020); *p-value < 0.05

Re-estimating the effect of dulaglutide on the MACE outcome after accounting for change in HbA1c, the observed hazard ratio changed from 0.88 to either 0.90 (95% CI 0.80, 1.01) or 0.92 (95% CI 0.82, 1.04) when the change from baseline or the updated mean was used, respectively (Table 1). Corresponding proportional mediation effects were calculated to be 16.7% and 36.1%, respectively. Similarly, after accounting for the change in UACR, the observed hazard ratio of dulaglutide vs placebo on the MACE outcome changed to 0.91 (95% CI 0.80, 1.04) and 0.91 (95% CI 0.80, 1.04) for the change from baseline and the updated mean, respectively, with proportional mediation effects calculated to be 25.4% and 28.5%, respectively (Table 1).

The change in both HbA1c and UACR over time was included in multivariable Cox models to estimate the degree to which the effect of dulaglutide on both variables could together statistically account for the effect on the MACE outcome. As noted in Table 2, the observed hazard ratio of dulaglutide vs placebo on the MACE outcome after statistically accounting for the updated mean value of both HbA1c and the UACR was 0.957 (95% CI 0.838, 1.093) with a mediation effect of 65.4%. When changes from baseline for both values were included in a Cox model, the observed hazard ratio for the effect of dulaglutide vs placebo on MACE was attenuated to 0.929 (95% CI 0.815, 1.060), with a mediation effect of 41.7%. There was no evidence of an interaction between the change in HbA1c and UACR for either model (P interaction = 0.75 and 0.15, respectively).

**Table 2:** Biochemical measurements that mediated the effect of dulaglutide on MACE-multi factor analysis

	HbA1c (%) and UACR (mg/mmol)
HR^a^ (95% CI) of dulaglutide on MACE (after adjusting for the baseline and updated mean value of the variable)	0.957 (0.838, 1.093)
Percentage mediated by updated mean towards the effect of dulaglutide on MACE^a^	65.4
HR^a^ (95% CI) of dulaglutide on MACE (after adjusting for the baseline and change from baseline value of the variable)	0.929 (0.815, 1.060)
Percentage mediated by change from baseline towards the effect of dulaglutide on MACE^a^	41.7

*CI* confidence interval; *HR* hazard ratio, *MACE* major adverse cardiovascular event, *UACR* urine albumin/creatinine ratio

^a^Calculated from a Cox proportional hazards regression model using time-dependent explanatory variables

## Discussion

This exploratory mediation analysis of the REWIND trial showed that the change in HbA1c was associated with 17–36% mediation on dulaglutide’s effect on MACE, and the change in UACR was associated with 25–29% on dulaglutide’s effect on MACE. It also showed that changes in both HbA1c and UACR together potentially mediated 42–65% of dulaglutide’s effect on MACE, and that HbA1c and UACR acted independently of each other. The mediation proportions that were calculated take into account the difference between the adjusted and unadjusted HRs, where the unadjusted HR remains the same as the HR for the primary endpoint reported in the original REWIND trial. The adjusted HRs are calculated separately by adjusting for various factors in the model and they are exploratory in nature. Therefore, any clinical or physiologic inferences will need further evaluation.

The only other existing analysis using the mediation analysis approach in GLP1- receptor agonists outcome trials on CV outcome was an exploratory analysis of the LEADER trial, which also investigated potential mediators to identify the effect of liraglutide on MACE [1]. The potential mediators investigated were HbA1C, body weight, UACR, confirmed hypoglycemia, sulfonylurea use, insulin use, systolic blood pressure, and LDL cholesterol. That analysis identified both HbA1c and UACR as potential mediators for effects on MACE in patients with type 2 diabetes using Cox models. The estimated effects were 10–41% for HbA1c and 22–29% for UACR, values similar to our findings. Also consistent with our analysis, body weight, SBP, and LDL were not found to be mediators for MACE reduction.

Our multivariable analysis found that changes in HbA1c and UACR together might mediate 42–65% of the effect of dulaglutide on MACE outcomes. This association could be due to the changes in these variables themselves, or more likely to changes in underlying biologic mechanisms that were reflected by these variables. Candidate mechanisms for HbA1c include direct effects of glucose on vasculature as well as indirect metabolic effects on cardiovascular disease [26]. Recent meta-regression analyses demonstrating a relationship in the degree of glucose lowering with GLP-1 receptor agonists and cardiovascular benefit provides some support for a direct glucose effect [27]. Changes in UACR likely reflect concomitant changes in endothelial function and renal function that may in turn be mediating the cardiovascular benefit [28], but independent of mean systolic blood pressure.

Neither our analysis nor the mediation analysis of the LEADER trial [1] identified other potential mediators. This may be due to either the size of the effect on these potential mediators (for example, LDL changed only 0.05 mmol/L) and/or the possibility that these changes are not related to the cardiovascular benefit of dulaglutide or liraglutide. Even with the mediation of HbA1c and UACR combined in the multi-factor model accounting for 65% (for the updated mean) of the effect of dulaglutide on MACE outcome, there still remains an unexplained percentage of the effect of dulaglutide.

Strengths of our findings include the long follow-up (median follow-up of 5.4 years), high study retention (97%), and high dulaglutide adherence (82%) in REWIND [15]. In addition, the MACE outcome was ascertained and adjudicated using the highest standards. Limitations include the fact that this is a post hoc exploratory analysis and therefore should be considered hypothesis-generating rather than providing definitive observations. Although we applied a widely accepted method of mediation analysis, any mediation analysis can only support (but not prove) the hypothesis that the identified mediators or factors linked to them are causally linked to the outcome. As noted above, although changes in HbA1c and UACR appeared to partly mediate the beneficial effects of dulaglutide, it is not known how these observations relate to the actual mechanism of benefit. Nonetheless, these findings add to the evolving body of literature reporting on mediators of MACE in patients with type 2 diabetes treated with GLP-1 receptor agonists.

In conclusion, the results of these analyses suggest a substantial portion of the benefits of dulaglutide on MACE may be associated with reduction of HbA1c and UACR. These effects appear to be additive, with up to 65% of the benefit statistically explained by these variables together, although residual mediators of benefit of dulaglutide remain unexplained.

## Data Availability

Eli Lilly and Company provides access to all individual participant data collected during the trial, after anonymization, with the exception of pharmacokinetic or genetic data. Data are available to request 6 months after the indication studied has been approved in the US and EU and after primary publication acceptance, whichever is later. No expiration date of data requests is currently set once data are made available. Access is provided after a proposal has been approved by an independent review committee identified for this purpose and after receipt of a signed data sharing agreement. Data and documents, including the study protocol, statistical analysis plan, clinical study report, blank or annotated case report forms, will be provided in a secure data sharing environment. For details on submitting a request, see the instructions provided at www.vivli.org.

## Abbreviations

CV: Cardiovascular
GLP-1: Glucagon-like peptide-1
HbA1c: Glycated hemoglobin
LDL: Low density lipoprotein
MACE: Major adverse cardiovascular events
SPB: Systolic blood pressure
UACR: Urine albumin/creatinine ratio
WHR: Waist-to-hip ratio
